# Miscellany

**Published:** 1909-04

**Authors:** 


### MISCELLANY.

---

Scientific Dietist.—Philippine Service.—The United States Civil Service Commission announces an examination on April 14, 1909, to secure eligibles from which to make certification to fill a vacancy in the position of scientific dietist, $900 per annum, for duty in the Philippines, and vacancies requiring similar qualifications as they may occur there. Board and quarters are furnished to the occupant of this position. Competitors will not be assembled for any of the tests. The examination will consist of the subjects mentioned below, weighted as indicated:

| | SUBJECTS. | WEIGHTS. |
|---|---|---|
| 1. | Thesis of not less than 500 words on a subject relating to dietics (to be submitted with application) | 25 |
| 2. | Experience (rated on application Form 375) | 75 |
| | Total | 100 |

Applicants must show in their applications that they have had extended experience in supervising and directing the formulating, preparing and serving of dietaries suitable to the needs of invalids or convalescents, such experience to have been acquired in an executive capacity in hospitals or similar institutions. Both men and women will be admitted to this examination. Age limits, 20 to 40 years on the date of the examination.

No application will be accepted unless properly executed and, with the material required, filed with the Commission at Washington prior to the hour of closing business on April 14, 1909. In applying for this examination the exact title as given at the head of this announcement should be used in the application.

---

The Messina earthquake is to be effectively described in the forthcoming *Century*. Mr. Robert Hichens, the novelist, will present a narrative of his observations and experiences during and after the catastrophe, and Professor Perret, who has been an assistant in the observatory of Vesuvius, has contributed a scientific account of it.

